# Why Chain Length of Hyaluronan in Eye Drops Matters

**DOI:** 10.3390/diagnostics10080511

**Published:** 2020-07-23

**Authors:** Wolfgang G.K. Müller-Lierheim

**Affiliations:** CORONIS Foundation, 81241 Munich, Germany; ML@coronis.net

**Keywords:** hyaluronan, hylan A, molecular weight, rheology, viscosity, eye drop, drug vehicle, homeostasis, pathophysiology of OSD, epithelial barrier

## Abstract

The chain length of hyaluronan (HA) determines its physical as well as its physiological properties. Results of clinical research on HA eye drops are not comparable without this parameter. In this article methods for the assessment of the average molecular weight of HA in eye drops and a terminology for molecular weight ranges are proposed. The classification of HA eye drops according to their zero shear viscosity and viscosity at 1000 s^−1^ shear rate is presented. Based on the gradient of mucin MUC5AC concentration within the mucoaqueous layer of the tear film a hypothesis on the consequences of this gradient on the rheological properties of the tear film is provided. The mucoadhesive properties of HA and their dependence on chain length are explained. The ability of HA to bind to receptors on the ocular epithelial cells, and in particular the potential consequences of the interaction between HA and the receptor HARE, responsible for HA endocytosis by corneal epithelial cells is discussed. The physiological function of HA in the framework of ocular surface homeostasis and wound healing are outlined, and the influence of the chain length of HA on the clinical performance of HA eye drops is illustrated. The use of very high molecular weight HA (hylan A) eye drops as drug vehicle for the next generation of ophthalmic drugs with minimized side effects is proposed and its advantages elucidated. Consequences of the diagnosis and treatment of ocular surface disease are discussed.

## 1. Introduction

The medical community owes highly purified hyaluronan to Endre A. Balazs who in the late 1960s found that hyaluronan did not cause inflammatory reaction in the owl monkey eye, and could be used to replace the pathological synovial fluid in arthritic joints, as well as the vitreous and aqueous humor of the human eye [1]. In 1976 the Swedish drug company Pharmacia took over the manufacturing and worldwide marketing of the highly purified, high molecular weight hyaluronan (HMW HA) under the trade name Healon (Pharmacia, Uppsala, Sweden) for use as therapeutic agent for pain relief in arthritis, and later for ophthalmic surgery. Robert C. Stegman was the first ophthalmic surgeon who in 1978 successfully injected Healon into the anterior chamber of human eyes to prevent corneal endothelium damage during cataract surgery [2]. The first report on the clinical use of hyaluronan eye drops in patients with severe dry eyes dates back to 1982. Polack and McNiece utilized a 0.1% HA solution from the remains of Healon syringes used for cataract surgery to treat patients with severe keratoconjunctivitis sicca [3]. Until the mid-1990s hyaluronan was believed to achieve its physiological functions exclusively by unspecific interactions such as lubrication, mechanical buffering, water homeostasis and macromolecular filtering [4].

Consequently, the Drug/Device Borderline Issues Working Group of the European Commission decided during their meeting on 24 and 25 October 1994 that hyaluronan containing products for medical application are regulated as medical devices, unless the manufacturer intends to claim pharmacological, immunological or metabolic activities as their main intended mode of action. Since that time this decision also includes hyaluronan eye drops as tear substitutes, provided that they do not achieve their principle intended action by pharmacological, immunological or metabolic means, but they may be assisted in their function by such means (see https://ec.europa.eu/docsroom/documents/35582). Since 1998 a plethora of hyaluronan eye drop brands containing different molecular weight hyaluronan in various concentrations have been approved as medical devices in Europe.

In 1995 the hyaluronan eye drops brand Hyalein of the Japanese ophthalmic drug manufacturer Santen (Osaka, Japan) received approval as a prescription drug for dry eye treatment by the Japanese authorities. These eye drops have proven their effectiveness as a highly biocompatible substitute for the aqueous phase of human tears. Whereas, eye drops obtained by dilution of Healon contain HMW HA, Hyalein eye drops contain low molecular weight hyaluronan (LMW HA).

In the United States tear substitutes are regulated as ”ophthalmic demulcents”, which, if containing certain active ingredients in established concentrations can be registered as OTC medicines without requiring a diligent approval process [5]. As hyaluronan is not on this grandfather list of active ingredients, no tear substitutes with hyaluronan as their sole active ingredient have so far achieved approval as an “ophthalmic demulcent” by the US Food and Drug Administration.

In 2017 the Tear Film and Ocular Surface Society (TFOS) published the results of the second Dry Eye Workshop (DEWS II) in a number of consensus papers on the definition, diagnosis, pathophysiology and treatment of dry eyes [6,7,8,9,10,11,12,13,14,15]. DEWS II defined dry eye as “a multifactorial disease of the ocular surface characterized by a loss of homeostasis of the tear film, and accompanied by ocular symptoms, in which tear film instability and hyperosmolarity, ocular surface inflammation and damage, and neurosensory abnormalities play etiological roles.” The concept of disruption of tear film homeostasis is considered to be the unifying characteristic of dry eye disease (DED), which is then classified with respect to presence or absence of symptoms (symptomatic versus asymptomatic), and with respect to etiology (aqueous deficient versus evaporative dry eye) [7]. DEWS II suggests a staged treatment of dry eye in which ocular lubricants (artificial tears) play a central role, and are considered to replace or supplement the natural tear film without targeting the underlying pathophysiology of DED [12]. Hyaluronan eye drops are offered as an option to increase tear viscosity and enhance lubrication due to their non-Newtonian shear-thinning properties [12].

Contrary to the DEWS II consensus, the Asia Dry Eye Society (ADES) restricts the definition of dry eye to patients having symptoms of either discomfort or visual disturbance [16]. ADES recognizes that hyperosmolarity is not necessarily a precondition of dry eye, but frequently a consequence of reduced blinking frequency, for example, in video display terminal (VDT) workers, and inflammation develops as a consequence in these patients [17]. Coherently the ADES defines dry eye as a ”multifactorial disease characterized by unstable tear film causing a variety of symptoms and visual impairments, potentially accompanied by ocular surface damage” [16]. The ADES classifies dry eye into three categories: aqueous deficient, increased evaporation and decreased wettability, recognizing that a compromised glycocalyx of the apical epithelial cells of the cornea will result in decreased water binding and lubricating properties of the ocular surface [18]. Secretory as well as membrane bound mucins are believed to play a major role in tear film instability, and there is clinical evidence that this underlying mechanism may be ameliorated by topical secretagogues such as diquafasol and rebamipide eye drops. Out of this consideration the ADES developed a tear film oriented treatment (TFOT) strategy, where hyaluronan eye drops are indicated for the treatment of aqueous deficient dry eye, and secretagogue eye drops may be prescribed for all of the three dry eye categories [18].

Recently published clinical results seem to indicate that very high molecular weight hyaluronan eye drops do not only act as water binding lubricants, but may actively target the underlying pathophysiological mechanisms of ocular surface disease [19,20]. In the following the current understanding of ocular surface homeostasis will, therefore, be summarized, and the molecular weight dependent physical properties of hyaluronan eye drops be discussed, proposals for terminology of molecular weight and classification of hyaluronan eye drops will be provided, and the concept of mucoadhesion based on entanglement between hyaluronan and mucus molecules will be illuminated. Subsequently the so far underestimated role of the extracellular matrix (ECM), and in particular hyaluronan, in favor of ocular surface homeostasis will be illuminated; the controversial clinical results with hyaluronan eye drops will be presented and explained. The extraordinary potential of the use of hylan A eye drops as a vehicle for active pharmaceutical ingredients (API) will be introduced. The consequences of the molecular weight dependent interaction of HA with the structures of the ocular surface for the diagnosis and treatment of ocular surface disease, as well as the need for future clinical research will be discussed.

## 2. The Current Understanding of Ocular Surface Homeostasis and the Role of Hyaluronan Lubricant Eye Drops

### 2.1. Ocular Surface Homeostasis

The healthy ocular surface epithelium is topographically smooth. The lipid bi-layer plasma membrane of the apical corneal epithelial cells is textured by microplicae which are lined with an antiadhesive, water binding, protective glycocalyx. The glycocalyx mainly consists of membrane bound mucins. It is covered by a mucoaqueous tear layer with lubricating properties predominantly due to dissolved gel-forming mucin MUC5AC secreted by conjunctival goblet cells [21,22,23,24,25,26,27,28,29]. The glycoprotein MUC5AC consists of a long protein molecule with oligosaccharide side chains linked by *O*-glycosylation to the protein backbone [23,30]. MUC5AC is an oligomer consisting of monomer units with an average molecular mass of 2.2 MDa [31]. The monomer units are linked by disulphide bonds resulting in the formation of MUC 5AC macromolecules with an average molecular weight of more than 40 MDa, forming very long linear flexible threads in excess of 10 μm length [23,31,32]. MUC5AC in the pre-corneal tear fluid was found to be of lower average molecular weight [33]. However, this finding may have been influenced by the sampling technique, and by the fact that the chain length of MUC5AC may not be evenly distributed within the pre-corneal mucoaqueous tear layer [34]. The flow behavior of dissolved long linear molecules (polymers) is determined by their ability to entangle [35]. Such polymer solutions exhibit viscoelastic, shear thinning characteristics as reported by Kaura and Tiffany for the tear film [36,37,38,39]. The tear film requires for stability reasons a high viscosity at rest (zero shear viscosity η_0_) and during blinking a low viscosity to prevent excessive shear stress on the ocular surface epithelia. For pooled tears of many normal human subjects Tiffany found a viscosity η’ = 65.5 mPa·s at low shear rate and η’ = 10.1 mPa·s at high shear rate [38]. Shear rate is defined as the speed of relative movement of two surfaces, for example, cornea and lid rim, divided by their distance, and therefore has the dimension s^−1^. The shear rate to which human tears are exposed is 0 s^−1^ in the open eye and typically 1000 s^−1^ during blinking.

Dry eye conditions are changing the rheology of tears [37]. Every form of dry eye, but also atopic keratoconjunctivitis, is associated with ocular inflammation, loss of goblet cells and reduced levels of MUC5AC [23,28,40,41,42,43,44,45]. The decrease in concentration, glycosylation or molecular weight of MUC5AC is associated with a disproportionate decrease of lubricating efficacy of the tear film. Elevated friction between the cellular structures of the ocular surface has been recognized as the driving force not only in dry eye disease (DED), but in all forms of ocular surface disease (OSD) [46]. Increased friction between the ocular surface tissues can originate not only from inadequate MUC5AC quantities dissolved in the mucoaqueous tear layer, but also from a compromised glycocalyx of the apical epithelial cells or elevated eye lid pressure [47]. The glycocalyx contains mucins bound to the plasma membrane of the apical epithelial cells of cornea and conjunctiva. The largest of these mucins, MUC16 extends 200–500 nm from the apex of microplicae into the mucoaqueous tear layer and prevents cellular adhesion as well as bacterial adherence and invasion [48,49,50,51]. Dry eye disease alters the degree of O-glycosylation in particular of MUC16, resulting in compromised wettability, water retention capacity, anti-adherence and lubricating properties, cellular surface barrier and eventually even dry spots on the epithelial surface [23,24,51,52,53,54,55,56]. It is worthwhile noting that MUC16 plays not only an important role for the cellular epithelial barrier function, but also contributes to the tight junctions between epithelial cells and thus for the paracellular barrier function [51]. Topographical irregularities of the ocular surface have recently drawn attention as an additional source of ocular surface friction [57]. The concentration and chain length of MUC5AC in the mucoaqueous layer may either be too high, causing mucous strands, blurring and high friction during blinking or too low resulting in insufficient lubrication and instable tear film [45]. It may be hypothesized that in aqueous deficiency the down-regulation of MUC5AC secretion initially contributes to preserving the homeostasis of the tear film. This assumption is consistent with the observation of Argüeso and colleagues who reported a significantly lower number of RNA transcripts for the goblet cell specific MUC5AC in the tear fluid of patients with Sjögren syndrome [41]. Dogru and colleagues reported that patients with atopic keratoconjunctivitis have a significantly reduced MUC5AC synthesis in combination with upregulated synthesis of membrane bound mucins presumably to protect the surface from mechanical damage [42,43]. Any persisting elevated friction during blinking exercises mechanical stress resulting in epithelial damage. This is visible as corneal and conjunctival staining and lid wiper epitheliopathy, inflammation of the lid rim eventually associated with obstruction of Meibomian gland orifices, and possible changes of ocular surface topography, like lid-parallel conjunctival folds (LIPCOF) or sandbank epitheliopathy [58,59,60]. In fact, only 60 s of eye rubbing are sufficient to cause elevated levels of inflammatory markers like MMP-13, IL-6 and TNF-α in healthy eyes [61]. It can therefore be assumed that permanent stress on the ocular epithelial tissues due to elevated friction triggers an acute inflammatory response and may finally cause chronic inflammation. In material science the term fatigue is used to describe the weakening of a material caused by cyclic mechanical loading [62]. While the influence of repeated mechanical stress on biological materials has been studied in the past mainly in dentistry and orthopedics, its influence on soft tissues with a high rate of turnover like the epithelia covering the ocular cornea and conjunctiva has only recently caught attention. Van Setten coined the term “attrition” for the cyclic mechanical stress by friction exercised by the lid to the underlying epithelial tissues during blinking and identified attrition as a potential cause for ophthalmic surgery failure [63]. Al-Aqaba and colleagues described the potential role of attrition of corneal nerves in neurotrophic keratopathy, post-penetrating keratoplasty, laser refractive surgery and chronic corneal edema, and concluded that attrition might also explain the observed lack of correlation between corneal epithelial nerve density and corneal sensitivity [64]. Intermittent unidirectional shear stress on corneal epithelial cells upregulates the formation of tight junctions between the cells [65]. There is evidence that mechanical stress to soft biological tissues may induce electric polarization [66]. In a recent review van Setten investigated the current knowledge of the potential role of attrition and intercellular shear in dry eye disease [67]. Attrition should be kept in mind as a factor of potential importance in understanding the influence of friction on ocular surface disease.

During the intervals between blinking the osmolarity of the mucoaqueous tear layer gradually rises due to water evaporation, which increases salt concentration. Osmolarity homeostasis is one of the major regulatory mechanisms of the entire human body [68,69,70]. Therefore, the osmolarity of the aqueous tear phase secreted by the major tear glands is well regulated. The peak value of osmolarity rise between two blinks mainly depends on the thickness of the tear layer, the time interval between blinks and the water retention properties of glycocalyx and tears [8]. Hyperosmolarity of the mucoaqueous tear layer has been suggested as one of the major etiological factors in ocular surface inflammation [71,72,73,74,75]. The innervation of the corneal epithelium includes cold thermoreceptors sensitive to changes in temperature and osmolarity through TRPM8 channels [76]. Increases in extracellular osmolarity or temperature decreases of the tear film due to evaporation trigger the eye blinking [77]. Blinking in turn results in redistribution of tear film from the tear reservoir of the tear meniscus.

Ocular surface irregularities are causing localized areas of thin tear layer. Intensive viewing as experienced by video display workers causes prolonged time intervals between blinking and thus higher fluctuations of osmolarity between blinks [78,79]. The lipid layer covering the mucoaqueous phase has for decades been believed to be the most important barrier against tear evaporation, and thus Meibomian gland disease (MGD) to be the most frequent underlying etiology of dry eye disease (DED) [7,80,81]. However, there is recent evidence that the thickness of the tear lipid layer has only negligible influence on the tear evaporation rate [82,83,84]. The prevailing belief that the tear film in DED is compromised due to increased evaporation as a consequence of a defective lipid layer has therefore been questioned [85]. On the other hand gel-forming mucins like MUC5AC in the mucoaqueous layer of the tear film and the mucins of the glycocalyx of the apical epithelial cells at the ocular surface have the ability to retain water through their carbohydrate side-chains [8,9,44,56,86]. Dry eye is associated with reduced concentration of MUC5AC as well as reduced glycosylation of the MUC5AC and MUC16 molecules [23,24,40,41,52,87]. It seems likely that this is one of the major reasons for increased tear evaporation rate and thus for hyperosmolarity or fluctuating osmolarity in patients with ocular surface disease.

### 2.2. Physical Properties of Hyaluronan-Containing Eye Drops

Hyaluronan (HA, hyaluronic acid, sodium hyaluronate, natrii hyaluronas) is a member of the large family of glycosaminoglycans (GAG), which are the main components of the extracellular matrix (ECM) [88]. Unique features that distinguish HA from other GAG are its simple structure and large molecular size up to 10 MDa. From a physical point of view, ideal tear substitutes should mimic the flow characteristics of natural tears, they should have affinity to mucins, in other words, be mucoadhesive, and exhibit in addition a high water binding capacity to counteract evaporation of the tear film between blinking.

The properties of hyaluronan in eye drops depend on chain length (weight average molecular weight and dispersion) and concentration. Whereas, the concentration of HA is usually part of the labeling of the finished product, the labeling rarely contains any information about chain length. This makes it very difficult to correlate the performance of different products reported in the literature. Already in 1995 Shimmura and colleagues noted that “previous studies have evaluated the efficacy of sodium hyaluronate as a tear replacement in the treatment of dry eyes with various results” [89]. Some of the clinical studies had used very high molecular weight hyaluronan by diluting Healon [3,90,91,92,93,94], others used low molecular weight hyaluronan originating from Viscoat (Alcon Laboratories, Fort Worth, TX, USA) [95] or other sources [89], and some did not specify the source or average molecular weight of the hyaluronan at all [96,97]. In some studies the eye drops had been autoclaved prior to use which is known to decrease the average molecular weight of hyaluronan by hydrolysis [90,97], and one study used purified water as a diluent which results in a strongly hypotonic solution [96].

Methods employed in the determination of the weight average molecular weight of hyaluronan include viscometry, osmometry, sedimentation/diffusion, light scattering and gel permeation/size exclusion chromatography (GPC/SEC) [98,99,100,101,102,103,104,105,106,107,108,109,110,111,112,113,114,115]. Low angle light scattering (LALS) and multi-angle light scattering (MALS) allow the determination of the hyaluronan chain length. The combination of fractionation by GPC/SEC with LALS or MALS allows the assessment of the molecular weight distribution/polydispersity of a hyaluronan solution [116,117,118,119,120,121,122,123,124]. There are a number of possible sources of error in particular when analyzing solutions containing very high molecular weight hyaluronan (>2 MDa) by SEC/MALS. Shear degradation of the HA molecule and non-ideal SEC fractionation may occur making it favorable to replace fractionation by the size exclusion chromatography by asymmetrical flow-field flow fractionation (FFF) [116,117,125]. The use of the polydispersity index as a measure of the molecular weight distribution may be misleading [126]. The determination of the molecular weight by multi-angle laser light scattering may be subject to some inaccuracy [127]. Recently fractionation of high molecular mass hyaluronan by gel electrophoresis has been proposed as a means of molecular mass determination as well as calculation of weight-average and number-average values [128]. The availability of defined megadalton hyaluronan standards has improved the molecular weight estimation by conventional SEC [129,130].

So far only the method to determine the intrinsic viscosity [η] of hyaluronan has been standardized and published in the European and Japanese Pharmacopoeias [131,132]. Therefore, the specification of the intrinsic viscosity of hyaluronan in future publications on hyaluronan-containing eye drops is highly recommended. The value of the intrinsic viscosity depends on the salt concentration of the solvent [133]. Whereas, the European Pharmacopoeia (EP) refers to 0.15 M sodium chloride in 0.01 M phosphate buffer solution, the Japanese Pharmacopoeia (JP) requires 0.2 M sodium chloride solution. The differences in the intrinsic viscosity values determined with both solvents are insignificant [112]. The intrinsic viscosity [η] of linear polymers allows the calculation of the average chain length as average molecular mass M_rm_ by the Mark–Houwink equation

[η] = κ × (M_rm_)^α^

where the coefficient κ and the exponent α are specific for the polymer molecule and the solvent. Table 1 provides an overview of published combinations of κ and α for linear, not crosslinked HA dissolved in 0.1 to 0.2 M sodium chloride aqueous solution, where the dimension of [η] is m^3^/kg and of the average molecular mass M_rm_ is Dalton. It needs to be pointed out that the average molecular mass of hyaluronan calculated from the intrinsic viscosity depends on the choice of the Mark–Houwink coefficients, and that the actual rheological characteristics of hyaluronan eye drops, moreover, depend on the polydispersity of the hyaluronan and the exact composition of the eye drops including their osmolarity.

According to Rolf Bergman (private communication 2009) the best fit of the rheological characteristics of HA solutions over the entire range of intrinsic viscosity can be achieved by the following combination of coefficients:

κ = 0.13327 × 10^−3^ and α = 0.6691

The Japanese Pharmacopoeia contains a monograph on sodium hyaluronate ophthalmic solutions [134] which limits the intrinsic viscosity to

1.18 m^3^/kg < [η] < 1.95 m^3^/kg

In Europe there is no such limitation; HA with significantly higher intrinsic viscosity is used in eye drops. In order to make the results of clinical investigations comparable, the terminology presented in Table 2 is proposed for its use in future publications on hyaluronan containing eye drops.

The higher the HA average molecular weight the higher the water binding capacity and the longer the retention time on the ocular surface [136,137]. The influence of average molecular weight of HA on the viscoelastic properties of HA solutions has been extensively studied by Bothner, Waaler and Wik [110,138,139,140]. For ideal linear polymers the zero shear viscosity η_0_ is a function of the product of concentration c and weight average molecular weight M_rm_(see Equation (1)).

(1) $$\eta_{0}\;\sim \;{\left(c\;\times \;M_{\mathrm{rm}}\right)}^{k}$$

**Figure 1:**
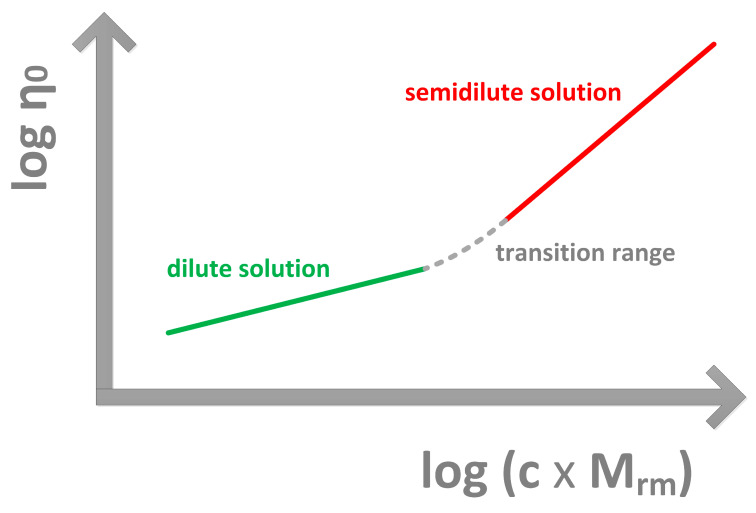
Illustration of the dependence of zero shear viscosity η_0_ from the product of concentration and average molecular mass (c × M_rm_) of solutions of an ideal linear polymer.

In dilute solutions of ideal polymers there is negligible interaction between individual polymer chains; these dilute solutions exhibit Newtonian flow characteristics with the exponent k = 1. Above a critical value of the product (c × M_rm_) the ability of the polymer chains to entangle determines the viscoelastic flow characteristics; for semidilute solutions of ideal polymers k = 3.4 [141,142]. Figure 1 illustrates the different ranges of flow characteristics of polymer solutions on a double logarithmic scale.

Bothner [139] found for hyaluronan molecules in aqueous solution the relationships provided in Equations (2) and (3)

η_0_ = 1.7 × 10^−7^(c × M_rm_)^0.74^  for the dilute region (η_0_ < 10 mPa·s) (2)

and

η_0_ = 5.3 × 10^−26^(c × M_rm_)^3.6^  for the concentrated region (η_0_ > 100 mPa·s) (3)

This means that, whereas, in the dilute region (low concentration of hyaluronan) doubling of molecular mass results in 1.67 times higher zero shear viscosity η_0_, in the semidilute region (medium to high concentration of hyaluronan) it results in 12 times higher zero shear viscosity. The longer the hyaluronan chains the more can they entangle and the more viscoelastic the solution is. In the assessment of hyaluronan eye drops the extent of their viscoelasticity can be characterized according to Equation (4) by the quotient Q of the zero shear viscosity η_0_ (= plateau of viscosity at very low shear rate) and the viscosity η_1000_ at 1000 s^−1^ shear rate (typical for the shear rate between lid rim and corneal surface during blinking)

(4) $$Q=\frac{\eta_{0}}{\eta_{1000}}$$

**Figure 2:**
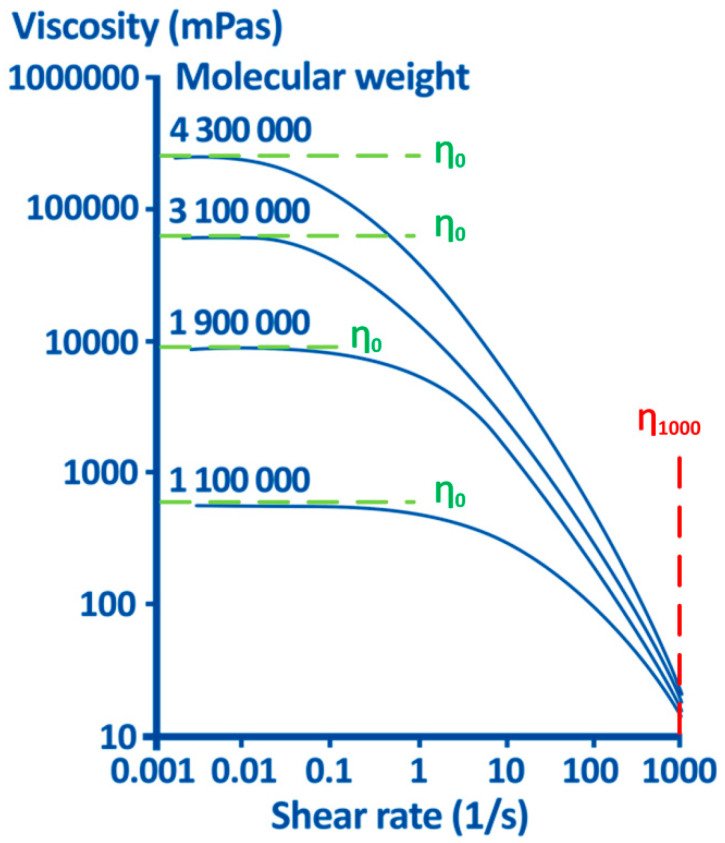
Dependance of the viscosity of 1% aqueous hyaluronan solutions from shear rate, modified from Bothner and Wik [138].

Q = 1 for Newtonian liquids like water. Bothner and Wik have measured the dependence of viscosity from the shear rate for different average molecular weight of hyaluronan in the semidilute range (1% HA) and confirmed the strong dependence of η_0_ from molecular weight as compared to the minor dependence of η_1000_ (see the red vertical broken line in Figure 2) [138].

**Figure 3:**
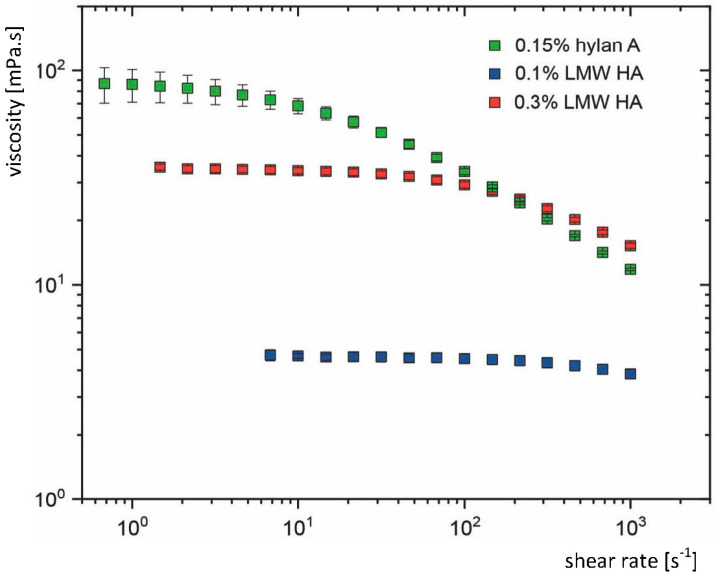
Flow characteristics of eye drops containing different average molecular weight and concentration of hyaluronan.

Only hyaluronan eye drops with a high quotient Q possess the potential to combine high viscosity in the open eye (long residence time on the ocular surface and thus effectiveness) with low viscosity during blinking (low friction to minimize shear stress during blinking, high ocular comfort and absence of blurred vision). The rheology (dependence of viscosity from shear rate) varies between the different commercially available HA eye drops. We measured the dependence of viscosity from shear rate of Hyalein Mini 0.1% and Hyalein Mini 0.3% from Santen, Osaka, Japan, both containing LMW HA, and Comfort Shield SD from i.com medical, Munich, Germany containing 0.15% very high molecular weight HA (hylan A). All three eye drops are isotonic, preservative free, and do not contain viscosity enhancing polymers other than HA. As could be expected, marked viscoelasticity was measured in the product containing very high molecular weight HA while viscoelasticity was rare or absent in the products with LMW HA (Figure 3). Q values for Comfort Shield SD were Q = 8 (η_0_ = 88 mPa·s; η_1000_ = 11 mPa·s), for Hyalein Mini 0.3% were Q = 2 (η_0_ = 35 mPa·s; η_1000_ = 17 mPa·s) and for Hyalein Mini 0.1%, Q = 1.25 (η_0_ = 4.8 mPa·s; η_1000_ = 3.8 mPa·s).

### 2.3. Hypothesis on the Rheological Characteristics of the Mucoaqueous Tear Layer

The flow behavior of the mucoaqueous tear layer is predominantly determined by the dissolved MUC5AC linear polymer chains. The glycoprotein MUC5AC consists of a long protein molecule with oligosaccharide side chains, similar to a bottle brush [24]. The chemical nature of the glycosaminoglycan hyaluronan is close to these oligosaccharides (glycans). It seems therefore justified to assume that the entangling properties of MUC5AC are roughly similar to those of very high molecular weight hyaluronan chains. In pooled tears from many human subjects, Tiffany found a viscosity η’ = 65.5 mPa·s at low shear rate and η’ = 10.1 mPa·s at high shear rate [38]. For hyaluronan solutions the transition range between dilute and semidilute flow behavior lies between 10 and 100 mPa·s [110]. The viscosity measured by Tiffany at low shear rate is likely to be on the viscosity plateau for low shear rate. This means that the zero shear viscosity of healthy human tears is about 65 mPa·s or slightly higher. Therefore, it is hypothesized that the zero shear viscosity of bulk tear fluid is in the transition range between a dilute and a semidilute solution, closer to the semidilute range. The recent understanding of a MUC5AC gradient within the mucoaqueous tear layer with higher MUC5AC concentration next to the glycocalyx of the apical epithelial cells suggests a reconsideration of the flow behavior of tears [8,22,23,143,144]. The flow characteristics of the mucoaqueous layer are likely to be that of a dilute polymer solution next to the lipid layer with a linear dependence of the zero shear viscosity η_0_ from the MUC5AC concentration c (Equation (5))

(5) $$\eta_{0}\;\sim \;c$$

whereas, the mucoaqueous layer next to the glycocalyx of the apical epithelial cells where the MUC5AC concentration is highest will show the characteristics of a semidilute polymer solution with exponential dependence of the zero shear viscosity η_0_ from the MUC5AC concentration c (Equation (6))

(6) $$\eta_{0}\;\sim \;c^{3.4}$$

**Figure 4:**
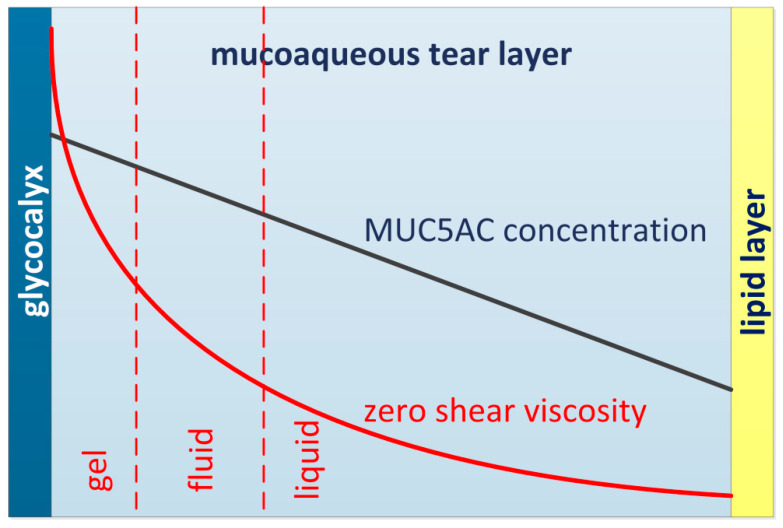
Illustration of the dependence of the zero shear viscosity of the mucoaqueous tear layer from the local MUC5AC concentration.

Above a critical concentration of MUC5AC, which is reached in the vicinity of the glycocalyx the extent of MUC5AC chain entanglement is intense enough to form a gel (see illustration in Figure 4).

The gel next to the glycocalyx acts as a mechanical cushion in addition to the lubricating effect of the part of the mucoaqueous layer closer to the surface. Most likely the exchange rate of the gel due to blinking is less than that of the remaining tear film [9]. Regions of high MUC5AC concentration — by some authors characterized as “blankets”— are capable of entrapping and removing cell debris, pathogens, allergens and particulate matter from the ocular surface and act as part of the ocular surface barrier [23]. Even minor variations of MUC5AC concentration, but also glycosylation or average chain length will strongly influence the viscosity at rest η_0_ of the mucoaqueous layer next to the glycocalyx of the epithelia where the concentration of MUC5AC is highest. This also explains the formation of mucous strands as a result of excess MUC5AC secretion. Assuming that the tear viscosity measured by Tiffany for high shear rate was representative for blinking and thus close to η_1000_, this would mean that the quotient Q of healthy tears would be 6.5 or even higher.

Tiffany in 1994 published viscosity measurement results for human tears between 65.5 mPa·s at low shear rate and 10.1 mPa·s at high shear rate [38]. Georgiev and Yokoi refer in their recent article on the physical properties of the tear film to shear dependent viscosity between 9 mPa·s at low shear rate and 1 mPa·s at high shear rate, which had been published by Tiffany and colleagues in 1998 [39,145]. It is likely that this obvious discrepancy in the tear viscosity values published by Tiffany has resulted from different sampling techniques [34]. Taking samples preferably from the superficial dilute phase of the mucoaqueous tear layer will lead to a significant underestimation of the viscosity of homogenized bulk mucoaqueous tear film.

### 2.4. Classification Proposal for Hyaluronan Eye Drops

As pointed out above, tear substitutes should mimic the flow characteristics of the mucoaqueous tear layer. This means that their zero shear viscosity should be close to that of bulk of healthy tears, in other words, η_0_ ≈ 65 mPa·s and their viscosity at 1000 s^−1^ shear rate η_1000_ no more than 13 mPa·s (i.e., 30% higher than human tears in healthy eyes) [146]. The categorization as outlined in Table 3 is proposed.

Endre A. Balazs coined the term hylan for cross-linked HA and added the letters A or B for different methods of cross-linking [147]. The letter A was reserved for “cross-linking” HA by formaldehyde [136,148,149,150]. Later Balazs confirmed that the use of formaldehyde applied in the preparation of hylan A did not result in cross-linking [135]. Numerous publications on eye drops refer to hylan A as “cross-linked” HA, but this needs to be corrected in favor of “very high molecular weight” HA. Recently eye drops containing crosslinked HA have become available. Little is known so far about the clinical performance of these eye drops. The globular structure of crosslinked HA molecules suggests that these molecules have less capability to entangle, and eye drops containing crosslinked HA are likely to exhibit less viscoelastic flow characteristics than those containing HMW linear HA molecules. Crosslinking reduces the effective chain length and flexibility of the HA molecule and thus reduces their mucoadhesive strength [151]. Moreover, the safety and pharmacological performance of crosslinked HA in eye drops will need further investigation.

### 2.5. Mucoadhesive Properties of Hyaluronan

Mucoadhesion is by definition the effect that two surfaces, one of which is a mucous membrane, adhere to each other [152]. Mucoadhesion has become a matter of interest because of its potential to optimize local drug delivery by retaining and releasing the active pharmaceutical ingredient (API) close to the site of action [151,152]. The theory of interaction between hydrogels and mucus has been extensively investigated by Peppas and coworkers [153,154,155,156,157,158]. Mucoadhesion occurs in two steps: interpenetration and tethering of a mucoadhesive polymer possessing functional groups like hydroxyl (OH), carboxyl (COOH), amide (NH_2_) or sulphate (SO_4_H) with mucin chains followed by hydrogen bond formation between the mucoadhesive polymer and the mucin chains [151]. When incorporated into a mucous gel or layer mucoadhesive polymers increase the resistance of the gel against deformation (termed rheological synergism) and strengthen the layer [159,160,161]. The flexibility of the polymer chains is important for interpenetration and entanglement with the mucin chains to allow the formation of hydrogen bonds [151,152]. Crosslinking within a polymer system significantly reduces chain motility and thus mucoadhesive strength [151,162]. Horvat and colleagues confirmed this for hyaluronan [163]. Mucoadhesive polymers may serve as drug carriers, but may also be used in their own right to coat and protect, for example, damaged tissues, or act as lubricating agents [152]. At the ocular surface mucoadhesion refers to the interaction with mucins of the mucoaqueous tear layer as well as with the membrane bound mucins of the glycocalyx of the apical epithelial cells. Hyaluronan is a highly flexible polyanion able to intimately entangle with and adhere to the mucin molecules at the ocular surface. Graça and colleagues recently summarized in vitro techniques to evaluate the mucoadhesive properties of hyaluronan-based ocular delivery systems [164]. They proposed determining the difference between the viscosity of a solution containing both mucin and hyaluronan and the added values of the viscosities of the solution of mucin and the solution of hyaluronan as a measure of mucoadhesive strength. Alternatively, they used a plate-plate rheometer, with pig eyes mounted on one of the plates, filled the space between the plates with solution, moved the plates with constant velocity and measured the force required to dissociate the sample from the probe. In a third assay they measured the zeta potential of mixtures of mucin and hyaluronan solutions. Khutoryanskiy discussed the validity and pitfalls of test methods for quantifying mucoadhesion [162]. Tensile tests measure the force that is required to detach a dosage form from mucosal tissue, for example, by using the dosage form as a substrate and fixing a pig eye on a mobile metallic probe. Tensile tests are rare because shear forces are likely to act on the vehicle [162]. In flow-through tests mucosal tissue is secured on the surface of a slide and covered by the mucoadhesive product to be tested. The product is washed off by rinsing with simulated body fluid, and the mucoadhesive polymer continuously measured in the perfusate. The exact experimental conditions including mucosal tissue, temperature, humidity and flow-rate of the eluting fluid are critical [162]. The rheological method described in detail by Hassan and Gallo, and also applied by Graça and colleagues monitors the forces in a mucoadhesive system by measurement of viscosity [164,165]. The interaction between the mucin molecules and the mucoadhesive molecules causes changes in the shape or arrangement of the macromolecules which are reflected in a change of viscosity [165]. Madsen and colleagues concluded from their experiments that the rheological synergism tested in rheological methods does not provide a complete explanation of the mucoadhesive phenomenon, and should not be considered as a stand-alone method to characterize mucus-polymer interactions [166]. Nevertheless, the rheological method is best suited to determining the interaction between mucus gel, such as MUC5AC and mucoadhesive polymers [162]. Hansen and colleagues reported the binding of high but not low molecular weight hyaluronan to cell membrane bound mucins strengthening the cellular barrier against pathogen invasion and extending the residence time for local drug delivery [167]. Whether the adherence of hyaluronan molecules to the apical surface of ocular epithelial cells is supported by mucoadhesion to membrane-bound mucins or rather interaction with receptors such as CD44 will need further investigation.

The introduction of thiol groups (SH) into mucoadhesive polymers results in so-called thiomers with the ability to form disulfide bonds between the polymer and cysteine-rich subdomains of mucus glycoproteins [168,169,170,171]. In the case of hyaluronan this is, however, likely to result in a loss of biological functionality and binding capacity to hyaladherins on the ocular surface.

### 2.6. Eye Drops Combining HA with Other Polymers

Some manufacturers of eye drops advocate mixtures of hyaluronan with other polymers to adjust the rheology of their tear substitutes [172]. This option is questionable for the following reasons. Very high molecular weight hyaluronan can substitute MUC5AC in the tear film and is absolutely non-allergenic. Moreover, very high molecular weight hyaluronan has the intrinsic ability to counteract inflammation [173]. The concept of a blur threshold of 20 to 30 mPa·s, which according to Aragona et al. is independent from the shear rate, is not supported by scientific evidence [172]. Tiffany found that healthy human tears exhibit extensive shear thinning properties with high viscosity (65.5 mPa·s) at low shear rate and low viscosity (10.1 mPa·s) at high shear rate [38]. Blurring is associated with blinking and thus with a high shear rate. Only the level of viscosity under shear stress is relevant for eye drops causing visual blurring. The more viscoelastic the eye drops are, the less blurring in combination with high zero shear viscosity can be expected.

## 3. The Physiological Role of Hyaluronan and the Extracellular Matrix in Ocular Surface Homeostasis

### 3.1. Physiological Activity of Hyaluronan in the Extracellular Matrix

The non-sulfated glycosaminoglycan hyaluronan occurs in multiple forms, chain length being the only distinguishing characteristic between them [174,175,176]. It may be free, bound to HA-binding proteins known as hyaladherins, or intercalated into complex structures such as the extracellular matrix (ECM) [177]. The balance between the production, sizing, secretion, and removal of HA are essential for its function in homeostasis and disease [178]. The synthesis of HA is assembled by three hyaluronan synthases, HAS1, HAS2 and HAS3, residing in the plasma membrane of virtually every cell and is simultaneously extruded into the extracellular space [179,180]. The three HA synthases produce HA of different chain length, the largest molecular weight HA being synthesized by HAS2. In the absence of inflammation most of the removal of HA occurs by metabolic degradation. In the blood stream about 85–90% of HA is eliminated by receptor-facilitated uptake and catabolism in the hepatic sinusoidal endothelial cells, moreover, 10% is extracted and catabolized in the spleen [181]. Lymph nodes also have a high capacity for extraction and catabolism of HA [181]. Zhou and colleagues identified two HA receptors in the liver and spleen, responsible for the endocytic clearance of HA and named them HARE (HA Receptor for Endocytosis) [182]. Banerji and colleagues identified the receptor responsible for the uptake and endocytosis of HA by lymph vessel endothelial cells and named it LYVE-1 [183]. Tammi and colleagues found that cultivated rat epidermal keratinocytes contain HA which is catabolized by lysosomal degradation intracellularly [184]. They found that the receptor CD44 is involved in the HA endocytosis by epidermal keratinocytes, but did not detect CD44 in the HA containing vesicles. Extracellular and intracellular HA showed a molecular mass of up to 6000 kDa and less than 400 kDa respectively, indicating that the keratinocytes selectively remove LMW HA from the extracellular matrix [184]. The long chain of HA molecules is very sensitive to cleavage by reactive oxygen species (ROS) as produced by inflammatory processes, but also by irradiation [185]. It seems likely that epidermal keratinocytes are capable to limit the proportion of LMW HA in the extracellular matrix in homeostasis.

Until the mid-1990s hyaluronan was believed to achieve its physiological functions exclusively by unspecific interactions such as lubrication, mechanical buffering, water homeostasis and macromolecular filtering [4]. The cloning of three HA cell surface receptors, CD44, RHAMM (receptor for HA mediated motility) and later layilin, triggered intensive HA research [186,187,188,189,190]. This finally led to our current comprehensive understanding of the active involvement of HA in development, tissue homeostasis, wound repair and inflammation.

In its high molecular weight form, HA has immunosuppressive effects [191,192,193]. Large HA molecules protect against lymphocyte-mediated cytolysis, suppress septic responses to lipopolysaccharides, maintain immune tolerance, induce production of immunosuppressive macrophages, reduce expression of inflammatory cytokines, modulate the immune system and are antiangiogenic; moreover, high molecular weight hyaluronan has intrinsic antiaging effects [173,174,191,194,195,196,197,198,199]. It has been postulated that particularly high molecular weight hyaluronan diminishes oxidative stress [88]. On the other hand it has been reported that reactive oxygen species (ROS) generated during inflammatory processes depolymerize HA and that the HA fragments trigger mucin MUC5AC hypersecretion [200]. Moreover, low molecular weight degradation products of hyaluronan can induce inflammation and angiogenesis [174,193,201,202]. Besides being mucoadhesive, hyaluronan can bind to a number of cell surface receptors (hyaladherins), including CD44, LYVE-1, HARE, layilin, TLR4, and RHAMM, thereby enabling temporal cell detachment and influencing cell proliferation, survival, motility and migration [178,203]. It has also been reported that HA is involved in neurogenesis [204].

Hyaluronan is present in all steps of the wound healing process, not only as an intrinsic component of the wound environment, but also as a factor that actively modulates tissue regeneration [88,205,206]. Low molecular weight HA is a product of hydrolysis of high molecular weight HA during inflammation, but, more importantly, acts also as a promotor of inflammation and of the entire process of wound repair [174,207]. Aya and colleagues therefore proposed bringing attention to the much-overlooked participation of HA in wound healing [174].

### 3.2. Molecular Weight Dependent Contribution of Hyaluronan to the Ocular Surface Homeostasis

Hyaluronan as well as its cell membrane bound receptor CD44 are co-localized in the basal cells as well as on the apical surface of the superficial epithelial cells of the cornea and conjunctiva [208,209,210,211]. The HA coating of the basal cells is consistent with the role of HA in promoting epithelial cell migration and proliferation [210,212,213,214,215]. Low concentration of HMW HA supports cross-bridging between the HA receptor sites of adjacent cells and may contribute to mechanical stabilization of the wing cell layers of the corneal epithelium [215,216,217,218]. An elevated level of CD44 and HA at the apical surface of the epithelium and the confirmed presence of HA in the tear film support the assumption that membrane bound HA may significantly contribute to hydration, lubrication and barrier function of the corneal epithelium and eventually even substitute compromised membrane bound mucins in the glycocalyx [210,219,220].

The molecular weight of membrane associated HA determines the activity of immune cells. Whereas, HMW HA (1.9 MDa) inhibits chemotaxis and phagocytosis of macrophages, LMW HA (300 kDa) hardly affects chemotaxis [221,222]. LMW HA is a potent activator of macrophages and induces IL-1β, TNF-α, and four members of the chemokine family [223,224]. HMW but not LMW HA inhibits directed neutrophil locomotion and binding of chemotactic factor to the neutrophil surface and neutrophil adhesion aggregation and binding to surfaces [225,226]. HA fragments are potent activators of dendritic cells [227,228]. The ability of HA to function as either a pro- or anti-inflammatory molecule is dependent on its size, microenvironment, localization, and availability of specific binding partners, and plays a role as crucial regulator of inflammation [173,229]. In the stroma of amniotic membrane HA plays an important role in the entrapment of inflammatory cells including lymphocytes [230]. On the other hand, reactive oxygen species (ROS) formed during inflammatory processes effectively degrades HMW HA which in turn supports the inflammatory process [231,232]. The metabolism of HA, therefore, deserves attention when considering the vicious circle of chronic inflammation in ocular surface disease [233].

HA plays a key role in corneal epithelial wound healing [88,203,234,235]. In this context it is of significance that the receptor HARE, responsible for HA binding and endocytosis, is strongly expressed on corneal epithelial cells [236,237].

High molecular weight, but not low molecular weight hyaluronan, has been shown to suppress activity in nociceptive afferent nerves, through the modulation of the polymodal transient receptor potential vanilloid subtype 1 (TRPV1) channel opening rate [238,239]. Moreover, HMW HA seems to be essential for the proliferation, differentiation and maturation of nerve cells [204]. These intrinsic properties of HMW HA are likely to contribute to the amelioration of symptoms in ocular surface disease and deserve further investigation.

## 4. Clinical Performance of Hylan A Containing Eye Drops in the Treatment of Ocular Surface Disease

Stegmann’s report on the first successful use of Healon in cataract surgery in 1978 [2] initiated the rapid spread of its use as a viscosurgical tool in ophthalmic surgery. Healon contains 1% of very high molecular weight hyaluronan (hylan A) dissolved in phosphate buffered saline solution. Excited about its universal performance in various types of surgery ophthalmologists soon found out that a 1:10 dilution of Healon provided alleviation of symptoms when instilled into the eyes of patients suffering from severe dry eyes [3,90,91,92]. Polack and McNiece originally had tried undiluted Healon, which relieved pain but became uncomfortable within minutes because of its high viscosity. After trying different dilutions they found out in a pretrial study that 0.1% HA concentration was preferred by most patients [3]. They tried this concentration on 20 patients with severe dry eye who had previously been using various artificial tear solutions as frequently as every 10 min. Their study included 15 patients with Sjögren syndrome, three patients with ocular pemphigus and two patients with Stevens–Johnson syndrome. Polack and McNiece reported that pain disappeared almost immediately after the application of hyaluronan drops and remained under control as long as the cornea was covered with the solution. Ocular redness improved within the first week of treatment without the use of steroids, and the improvement of iritis was related to the degree of epithelial healing. The hyaluronan eye drops appeared to adhere to the corneal epithelium, and Polack and McNiece observed by staining with fluorescein that the coating of the entire corneal surface lasted for at least an hour in most patients. DeLuise and Peterson confirmed the clinical performance of 1:10 diluted Healon in 28 Sjögren syndrome patients [90]. They reported a decrease in subjective discomfort and objective signs in 26 of 28 patients. In eight out of 23 patients who originally had mucus stranding the mucus strand formation diminished. Stuart and Linn reported about the use of 1:10 diluted Healon in 14 patients with keratitis sicca, two with corneal dystrophy, two with recurrent erosion, three with contact lens induced irritation, one with ocular pemphigoid, two with filamentary keratitis and two with neurotrophic keratitis [91]. Their patients had used the eye drops for up to two years. The patients with poorly controlled recurrent corneal erosions reported that attacks, although not eliminated, were not as disabling as before. “Two patients with severe keratitis sicca and rheumatoid arthritis presented with bilateral corneal perforation that required bilateral penetrating keratoplasty, and both have used 1:10 diluted Healon postoperatively for 13 and eight months with preservation of graft clarity”. Only one patient experienced burning sensation after the instillation of the eye drops and discontinued their use after one day. Mengher and colleagues reported on the use of 1:10 diluted Healon in 11 patients with keratoconjunctivitis sicca [92]. By measuring non-invasive break-up time they found significantly improved tear film stability. Moreover, symptoms of grittiness and burning were significantly alleviated. Wysenbeek and colleagues studied the protective effect of undiluted and 1:10 diluted Healon on the chick corneal epithelium by electron microscopy [93]; whereas, the application of 0.01% benzalkonium chloride (BAK) solution causes severe loss of microvilli and large holes in the cell membranes of some of the apical epithelial cells, the number of damaged cells is significantly reduced when applying 0.01% BAK dissolved in 1:10 diluted Healon. From their results it may be concluded that 1:10 diluted Healon exercises a protective effect on the epithelial cell membrane. This supports the hypothesis that very high molecular weight hyaluronan (hylan A) may either have a protective effect on the ocular surface epithelium by binding to the membrane bound mucins of the glycocalyx, in particular MUC16, or by binding to receptors like CD44, RHAMM or HARE at the ocular surface, thus supporting the wettability and stabilizing the barrier function of the corneal surface. Using the categorization proposed in Table 3, 1:10 diluted Healon falls into category 4.

To prove the hypothesis that hylan A is improving the wettability of and stabilizing the ocular surface epithelium, commercially available eye drops containing 0.15% hylan A (Comfort Shield, i.com medical, Munich, Germany) were tested by Kojima and colleagues [20]. This research group has established an animal model employing environmental dry eye stress (EDES) that mimics the air conditioning stress of visual display terminal work by exposing the animals for five hours daily over a period of three days to air blow from a fan. When exposed to EDES, mice develop inflammation mediated loss of tear volume and secretory Muc5AC, instable tear film resulting in decrease of tear break-up time (TBUT) and ocular surface damage. Kojima and colleagues compared 0.15% high molecular weight hyaluronan (HMW HA) eye drops (category 4 according to Table 3) with 0.1% low molecular weight hyaluronan (LMW HA) eye drops (category 1) and 0.3% LMW HA eye drops (category 5), as well as 3% diquafasol sodium (DQ) eye drops with respect to their effectiveness to prevent and treat EDES induced dry eye disease in mice. One hypothesis of the study was that due to their pronounced viscoelasticity HMW HA eye drops will stay on the ocular surface longer and provide improved lubrication, both resulting in a reduced staining score. Another hypothesis was that due the larger chain length the HMW HA molecules have a higher potential than LMW HA molecules to entangle with and substitute gel forming Muc5AC chains, which might result in improved tear film stability, and be reflected in prolonged tear film break-up time (TBUT). Moreover, if the HMW HA was to provide a significant anti-inflammatory effect on the ocular surface, this should result in the protection of the lacrimal glands and goblet cells against T cell-mediated damage, and be reflected in increased levels of tear volume and Muc5AC. The experiment comprised two phases of treatment with three drops daily of the respective eye drops: Three days of EDES exposure should show to what extent the application of eye drops can prevent the development of dry eye signs. The subsequent four days without EDES exposure were intended to study the effectiveness of the eye drops in supporting the healing of the ocular surface. Animals without eye drop application, both with and without EDES exposure served as positive and negative controls. At the end of the first period with three days ocular stress the aqueous tear volume of the animals treated with LMW HA was significantly decreased, whereas, the tear volume under treatment with hylan A or DQ remained unchanged. The tear film stability as determined by TBUT was decreased under LMW HA and DQ treatment, but remained unchanged under hylan A treatment. The surfaces of the eyes of the animals treated with LMW HA or DQ exhibited more staining with fluorescein and lissamine green than those treated with hylan A. By the end of the subsequent four-day recovery period, tear volume and TBUT of all animals had returned to normal, but the fluorescein and lissamine green staining scores of the ocular surface were still elevated in the LMW HA and the lissamine green staining score in the DQ treated animals, whereas all staining scores had returned to normal in the animals treated with hylan A. The mucin Muc5AC mRNA expression was significantly higher under hylan A treatment, indicating a better intrinsic lubrication efficacy of the animals’ own tears. Moreover, the density of infiltrated dendritic (inflammatory) cells was significantly lower after treatment with hylan A eye drops than under any other treatment [20]. These results underline the necessity to differentiate hyaluronan eye drops on the basis of their average molecular weight. The results of the animal study on the performance of hylan A eye drops in the prevention and treatment of environmental stress induced signs of dry eye support the hypothesis that hylan A eye drops do not only offer lubricating and water binding properties for the treatment of dry eye, but seem to act anti-inflammatory, stabilize the glycocalyx of the corneal epithelial cells, and protect the epithelium against mechanical damage. The observation that the tear volume under treatment with hylan A is equivalent and the Muc5AC mRNA expression even higher than with the well-established secretagogue DQ needs further investigation. Moreover, the extent to which hylan A eye drops support epithelial healing and ameliorate pain in dry eyes as reported by Polack and Niece needs further confirmation [3].

Recent clinical data on humans suggest that due to the molecular weight dependent physiological functions of hyaluronan the use of eye drops containing 0.15% hylan A is favorable in the therapy of severe OSD and in some cases may even be a substitute for autologous serum eye drops [19]. The results of a comprehensive multi-center study on the potential improvement of signs and symptoms of patients suffering from severe dry eyes by the use of 0.15% hylan A eye drops (HYLAN M study) will be published soon.

## 5. Hyaluronan Eye Drops as a Vehicle for Active Pharmaceutical Ingredients: Technology Platform for the Next Generation of Topical Treatment of Ocular Disease

Eye drops for the topical treatment of ocular diseases such as glaucoma, chronic inflammation, allergy and atopy are composed of an active pharmaceutical ingredient (API) with pharmacological, metabolic or immunological activity, dissolved or suspended in a vehicle. The vehicle has the tasks to dissolve or suspend the API, stabilize the solution during shelf-life of the eye drops and during patient use, prolong the contact time between the API and the ocular surface, support the penetration into the ocular surface, and enhance the biocompatibility of the eye drops [240,241].

Most eye drops are aqueous solutions requiring additives, in particular surfactants to dissolve lipophilic APIs. Eye drops need to be sterile; during patient use this can be achieved either by single-use containers (monodoses), bottles with particular dispensers preventing microbial contamination or by addition of preservatives like benzalkonium chloride. The contact time with the ocular surface can be prolonged by the addition of polymers increasing the viscosity of the solution. Mucoadhesive additives like hyaluronan can, moreover, adhere to the glycocalyx of the apical epithelial cells, thus promoting the contact between the API and the ocular surface. Penetration enhancers weaken the transcellular or paracellular epithelial barrier function thus enhancing the diffusion of the API into the ocular surface. Salts are added to adjust the osmolarity, and buffers to adjust and stabilize the pH value of the eye drops to a physiological level and to stabilize the eye drops.

Surfactants are capable to replace the cell-bound mucins in the glycocalyx of the apical epithelial cells and be incorporated in the lipid bilayer forming the cell membrane, and thus compromise the cell barrier function and support the transport of the API through the cell membrane into the cell [242,243,244]. Surfactants like benzalkonium chloride (BAK, cetalkonium chloride) and cationic polymers named polyquaternium are still widely used in ophthalmic drugs because they have a combined effect of dissolving the API in the aqueous solution, enhancing its penetration into the ocular surface and at the same time preserving the solution against microbial growth. These benefits are at the expense of local irritation and disastrous long-term ocular surface disease [245,246,247].

Additives like ethylenediamine tetra acetic sodium salt (EDTA) deprive the tight junctions between the epithelial cells from Ca^2+^ ions and thus weaken the paracellular barrier function of the epithelium.

Current eye drops for the treatment of glaucoma, as well as of chronic ocular inflammation cause serious side effects in a significant percentage of patients. These adverse ocular reactions are not only caused by the APIs, but to a large extent by the vehicles used. In clinical studies for regulatory approval and reimbursement the safety and performance of new topical ophthalmic drugs are frequently tested in comparison to the vehicle only. This strategy allows eliminating the negative effects of the vehicle, brightening the effect of the product.

The use of hyaluronan in ophthalmic drug vehicles has been suggested in numerous publications [242,248,249,250,251,252,253,254]. Hyaluronan has a proven effect of counteracting the irritating effect of substances to the ocular epithelium [93,255,256,257].

Hylan A contained as a vehicle in eye drops can not only stabilize the epithelial barrier function and act anti-inflammatory, but due to its viscoelastic and mucoadhesive properties promote the prolonged, intimate contact of APIs to the ocular surface [258]. Hylan A, but not low molecular weight HA, is binding to MUC16 in the glycocalyx of the apical epithelial cells [167]. Additionally, hylan A is known to strongly bind to HA receptors CD44, RHAMM and HARE present on the apical surface of the corneal and conjunctival epithelium. The affinity of binding HA to cell surface hyaladherins increases with HA chain length [217,259]. The ability of HA to bind to CD44 is dependent on interaction with multiple CD44 receptors [235]. The higher the molecular mass of HA the higher the avidity of the binding to CD44 [229]. Changing from weakly binding LMW HA to HMW HA results in binding with high affinity [260]. Cellular uptake of HA bound to the HARE receptor is likely to transport the API into epithelial cells by endocytosis without damaging the cell membrane [208,209,210,211,219,261]. Moreover, HA has been shown to transport nanoparticles across the epithelial barrier [262,263,264,265]. HA has also been reported to markedly enhance the efficacy of cyclosporine when injected intravenously [266]. The mechanisms of penetration enhancement by hylan A are not yet fully understood. Beside HARE mediated endocytosis another possible mechanism is that hylan A enables temporal cell detachment and thus enhances the penetration of nanoparticles and drugs along the paracellular pathway [88,174,178,203,214,266]. One of the physiological functions of HA is to contribute to local tissue hydration; this accounts for weakening of cell adherence to the ECM, allowing for temporal detachment that facilitates cell migration and division [203,215,267]. HA rich areas may separate physical structures and create “highways” for cell migration and transport of APIs [203,213,268]. Most likely high molecular weight hyaluronan is capable of transporting APIs across the epithelial barrier without compromising the ocular surface and its barrier function. The use of hylan A in drug vehicles will be associated with lower concentration of APIs to achieve the intended therapeutic effect; this will provide an additional reduction of the intrinsic side effects of the APIs.

Eye drops containing hylan A as vehicle have the potential of becoming the platform for the development of the next generation of topical ophthalmic drugs for the treatment of sight threatening diseases such as glaucoma and chronic ocular inflammation [269,270].

## 6. Discussion

Over the past 25 years, HA eye drops have gained recognition as the first choice of tear substitute in the treatment of dry eye disease (DED) in Europe and Asia. The consulting ophthalmologist has the choice from a plethora of HA eye drop brands. Tear substitutes are commonly understood as lubricant, viscosity enhancing, and water-retaining eye drops without targeting the pathophysiological processes of DED. Within this perception, the chain length of the HA molecules contained in these eye drops determines their viscoelastic and mucoadhesive properties, resulting in more or less entanglement and rheological synergism with the mucins dissolved in the mucoaqueous layer of the tear film, in particular MUC5AC, and mucoadhesive adherence to the membrane bound mucins in the glycocalyx of the apical epithelial cells of the cornea and conjunctiva. These physical properties of HA eye drops contribute to stabilize the tear osmolarity on the ocular surface and minimize the friction between the moving eye lid and the surface of the eye ball during blinking, thus reducing known stimuli of ocular surface inflammation. The stabilization of the mucins dissolved in the tear film by rheological synergism is also likely to strengthen the effectiveness of the gel forming mucin MUC5AC in removing particulates including allergens from the ocular surface.

It will also be of interest, if the pain related symptoms of dry eye patients will be ameliorated by the ability of HMW HA to suppress activity in nociceptive afferent nerves [238,239], and if there is an effect of HMW HA on the recovery of damaged corneal nerves. These questions deserve further clinical research.

The current focus in the diagnosis and treatment of chronic inflammatory ocular surface disease is directed at the involvement of lymphocyte dependent adaptive immune responses. However, there is growing evidence for the involvement of innate immune cells, and even tissue-resident stem cells can show adaptive characteristics [271]. Netea and colleagues have proposed the term “trained immunity” for the heightened response by innate cells to secondary infections both to the same microorganism and to a different one (cross-protection) [272]. They pointed to the potential detrimental outcomes that trained immunity may have in immune-mediated and chronic inflammatory diseases [271]. It may be of relevance to further investigate the role of trained immunity particularly in auto-immune diseases involving the ocular surface, and the interaction of hyaluronan eye drops with innate immune cells in these diseases.

None of the current diagnostic methods available in ophthalmological practice provide a measure for the viscoelastic properties of the tear film. Schirmer 1 and tear meniscus volume provide an idea on the aqueous tear production rate. The future simultaneous assessment of the MUC5AC secretion rate of the conjunctival goblet cells might allow an estimation of the rheological characteristics of the mucoaqueous tear film. Moreover, fluorometric tests to judge the quality of the ocular surface glycocalyx would be desirable for the routine diagnosis of ocular surface disease [273,274].

The ability of HMW HA molecules to strongly bind to CD44 and RHAMM receptors on the apical surface of ocular surface epithelial cells is likely to directly contribute to the lubricating, antiadhesive properties, and water retention of the glycocalyx of the apical corneal and conjunctival cells. It may be hypothesized that this is a protective mechanism independent from the established contribution of the membrane bound mucins.

The expression of the HARE receptor at the surface of corneal epithelial cells, which enables the HA uptake into the cytoplasm of these cells by endocytosis, has to my best knowledge so far not been taken into consideration as an essential factor contributing to the homeostasis of the ocular surface. Not only does this process enable corneal epithelial cells to control the HA concentration in their extracellular environment during wound healing and inflammation, but it may, moreover, be hypothesized that the enormous water binding capacity of HA within the cells will also protect corneal and conjunctival epithelial cells against dehydration.

Kojima and colleagues recently reported that hylan A containing eye drops, but not LMW HA containing eye drops preserve the tear volume and Muc5AC secretion under environmental stress in a mouse dry-eye model [20]. The most likely mode of action is the hylan A mediated protection of the lacrimal glands and goblet cells against T cell-mediated damage. This finding confirms the necessity to distinguish molecular weight of HA in eye drops. It is recommended that the distinction of molecular weight in HA eye drops will in the future also be reflected in the concept of tear film oriented therapy (TFOT) proposed by the Asian Dry Eye Society [16,18].

The finding by Beck and colleagues that hylan A containing eye drops can substitute autologous serum in patients with severe dry eyes, where other tear substitutes had failed to adequately ameliorate symptoms, sparks the question, whether or not HA eye drops might play a thus-far unconsidered, but important role in the pathophysiological processes underlying ocular surface inflammation. In the ongoing HYLAN M study, where only patients suffering from severe dry eyes with chronic inflammation were included, the control group continued to use their individual “optimum” tear substitutes, which they had been using before the time of inclusion, whereas, in the verum group these tear substitutes were replaced by hylan A containing eye drops. The results of this study may provide initial answers to the question whether hylan A containing eye drops are capable to target the pathophysiological processes of dry eye disease.

In recent years dysregulation of the epithelial barrier function has been recognized as a primary defect in the pathogenesis of atopy and allergic reactions [275,276]. It may be anticipated that hylan A containing eye drops will also prove their efficiency in the prevention and treatment of allergic ocular diseases by their supportive activity in mechanical removal of allergens from the tear film, their stabilizing contribution to the epithelial barrier function and their ability to suppress the activation of inflammatory cells [23,167,173,210,229,276]. However, particular attention needs to be taken when using HA eye drops in atopic and allergic patients, because while hylan A eye drops counteract inflammation, LMW HA eye drops may even promote inflammation.

Belmonte hypothesized that symptomatic differences in ocular surface disorders reflect differences in the balance between ocular inflammation and nerve injury [277]. He proposed to distinguish ocular surface disease according to the dominant pathophysiological mechanism evoked by the different etiologies of ocular surface disorders. Situations, where nerve injury is the dominant pathophysiological mechanism include surgical incisions for retinal and anterior segment surgery, photorefractive surgery, aging and peripheral neuropathies, for example, accompanying diabetes. Situations, where inflammation is the dominant pathophysiological mechanism include allergic keratoconjunctivitis, photokeratitis and all forms of acute eye surface inflammation, for example, accompanying video display work (VDT) and other environmental stress. In order to study the potential molecular weight dependent role of HA in the causative treatment of ocular surface disease future studies will need to focus on its interaction with nerve damage and recovery, as well as inflammation. Whereas, in clinical research inflammation has been mostly diagnosed by its reflection in the expression of cytokines and chemokines, as well as HLA-DR, in vivo confocal microscopy provides the possibility to simultaneously observe nerve fibers and inflammatory cells in all layers of the cornea.

The presence of HARE receptors at the surface of ocular epithelial cells allows them to internalize HA by endocytosis. This is a novel option to transport active pharmaceutical ingredients (API) with HMW HA molecules as vehicle through the cell membrane of epithelial cells without damaging the cell membrane. In combination with the ability of HA to ameliorate the negative effects of corneotoxic substances [93,255,256,257], it is anticipated that particularly patients requiring long-term topical treatment of diseases like glaucoma, who currently suffer from negative side effects of their treatment, will benefit from the new technology.

## 7. Conclusions

There are numerous publications on hyaluronan eye drops lacking the molecular weight of the biomolecule hyaluronan. Due to the molecular weight dependence of physical properties as well as pharmacological activity of hyaluronan, it is desirable to provide information on the intrinsic viscosity of the hyaluronan contained in eye drops, in order to render clinical results and claims comparable. A first step in this direction would be the classification of hyaluronan eye drops as proposed in Table 3. The clinical performance of eye drops containing very high molecular weight hyaluronan (hylan A) is entirely different from that of eye drops containing low to medium molecular weight HA. Further clinical studies on the role of hylan A containing eye drops in the regulation of ocular surface inflammation, corneal wound repair, regeneration of compromised nerves, immunoregulation, alleviation of the symptoms of allergic keratoconjunctivitis and atopy, as well as ameliorating pain related symptoms by interaction with the superficial ocular nerves are required. Little is known about the mechanisms by which hyaluronan protects the ocular surface against noxious agents like benzalkonium chloride. It seems likely that a better understanding of the influence of hyaluronan chain length in contact to the ocular surface on the clinical performance of eye drops will lead to an increased use of HMW hyaluronan eye drops in the treatment of ocular surface disease. HMW HA is supporting the transport of nanoparticles and drugs across the epithelial barrier of the eye. It is a candidate for the replacement of current penetration enhancers, and will significantly reduce side effects in long-term topical treatment of ocular diseases like glaucoma, allergy/atopy and chronic inflammation. Hylan A as a side-effect free vehicle, replacing penetration enhancers in current eye drop formulations has the potential to become the platform technology for the next generation of topical eye drops.

## Figures and Tables

**Table 1:** Published constants in the Mark–Houwink equation for HA [η] in units m³/kg).

Reference	M_rm_ Range	κ	α
Laurent et al. 1960 [104]	0.077–1.7 MDa	0.036 × 10^−3^	0.78
Cleland and Wang 1970 [105]	0.1–1.0 MDa	0.0228 × 10^−3^	0.816
Shimada and Matsumura 1975 [106]	0.31–1.5 MDa	0.057 × 10^−3^	0.76
Balazs et al. 1981 [109]		0.029 × 10^−3^	0.80
Bothner et al. 1988 [110]	0.1–1.0 MDa	0.0346 × 10^−3^	0.779
	>1.0 MDa	0.397 × 10^−3^	0.601
Ueno et al. 1988 [111]	0.25–1.63 MDa	0.039 × 10^−3^	0.77
Fouissac et al. 1992 [112]	>2.4 MDa	0.016 × 10^−3^	0.841
Yanaki and Yamaguchi 1994 [114]	0.4–2.66 MDa	0.0199 × 10^−3^	0.829
Soltes et al. 2002 [118]	0.42–1.38 MDa	0.0278 × 10^−3^	0.78
Japanese Pharmacopoeia XVII [132]	0.50–1.49 MDa	0.036 × 10^−3^	0.78
	1.5–3.9 MDa	0.0228 × 10^−3^	0.816

**Table 2:** Terminology proposed to define the relation between the hyaluronan average molecular weight ranges in eye drops and its intrinsic viscosity values.

Classification	Intrinsic Viscosity [η] (m^3^/kg)	M_rm_ According to JP (MDa)	M_rm_ According to Bergman (MDa)
LMW HA	<1.8	<1.05	<1.5
MMW HA	1.8–<2.5	1.06–1.4	1.5–2.3
HMW HA	2.5–<2.9	1.5–1.7	2.4–2.9
hylan A	≥2.9	≥1.8	≥3.0

LMW HA: low molecular weight hyaluronan; MMW HA: medium molecular weight hyaluronan; HMW HA: high molecular weight hyaluronan; hylan A: coined by Endre Balazs for very high molecular weight linear hyaluronan [135].

**Table 3:** Proposed categorization of HA eye drops based on their flow characteristics.

Category	η_0_ (mPa·s)	η_1000_ (mPa·s)
1	η_0_ < 6	η_1000_ ≤ 13
2	6 ≤ η_0_ < 16	η_1000_ ≤ 13
3	16 ≤ η_0_ < 32	η_1000_ ≤13
4	32 ≤ η_0_	η_1000_ ≤ 13
5	-	η_1000_ > 13
